# Role of Neck Imaging Reporting and Data System in Evaluation of Recurrent/Residual Lesions of Head and Neck Cancers by Contrast-Enhanced Computed Tomography (CECT) With Pathological Correlation

**DOI:** 10.7759/cureus.103321

**Published:** 2026-02-09

**Authors:** Vaishnavi Yeerasam, Aditi Nadamani, Suresh A, Mary Varunya

**Affiliations:** 1 Radiology, Vydehi Institute of Medical Sciences and Research Centre, Bengaluru, IND

**Keywords:** cect neck, head and neck ct, head and neck radiology, local recurrence of head and neck cancer, nirads

## Abstract

Introduction

Head and neck cancers remain a major global health concern, mainly involving the oral cavity, pharynx, larynx, thyroid, and salivary glands. The Neck Imaging Reporting and Data System (NI-RADS) has been developed as a structured framework in order to enhance the quality and consistency of imaging reports for patients undergoing surveillance after treatment for head and neck cancers. NI-RADS provides a standardized lexicon that simplifies the communication of imaging findings. This is crucial given the intricate anatomical and pathological changes that can occur following treatment, which often complicate the interpretation of imaging studies. In the context of head and neck cancer, the post-treatment landscape is characterized by a range of changes, including surgical alterations, radiation effects, and potential disease recurrence. Traditional reporting methods can lead to ambiguities, misinterpretations, and inconsistent management decisions. NI-RADS mitigates these challenges by categorizing findings into a systematic reporting structure that emphasizes clarity. Each category (NI-RADS 1 through NI-RADS 4) offers specific definitions and criteria that assist radiologists in assessing the likelihood of residual disease or recurrence, thus facilitating more informed clinical decision-making and improving patient outcomes. By providing a common language, NI-RADS helps bridge the gap between imaging interpretation and clinical management, ultimately enhancing the overall quality of care for patients with head and neck cancers.

Methods

Contrast-enhanced computed tomography head and neck images of 64 post-treatment cancer patients were evaluated and allotted a NI-RADS category for the primary site and lymph node involvement using the NI-RADS lexicon, and consequently followed up with clinical correlation and histopathological report to determine the sensitivity, specificity, negative predictive value, and positive predictive value.

Results

The majority of cases were classified as NI-RADS 1 (36 cases), followed by NI-RADS 3 (21 cases), and the least were NI-RADS 2 (7 cases). The overall recurrence rate at the primary site was 21.88% (14/64 cases). A strong correlation was observed between NI-RADS categorization and recurrence rates. NI-RADS 1 demonstrated a 0% recurrence rate, confirming its high negative predictive value (100%). It was also observed that NI-RADS 3 is highly effective in detecting recurrences, with a positive predictive value of 61.9% for the primary site and 100% for nodal recurrence. The findings also suggested that NI-RADS 2 remains a gray zone, where careful evaluation and further diagnostic steps are necessary. The lower positive predictive value (33.33%) of NI-RADS 2 for nodal recurrence and the recurrence rate at the primary site (14.3%) indicate that some cases categorized as NI-RADS 2 may progress to recurrence, necessitating individualized follow-up strategies.

Conclusion

This study reinforces the utility of NI-RADS as a reliable tool for risk stratification in head and neck cancer surveillance. The high statistical significance in both primary site and nodal recurrence underscores its predictive accuracy. NI-RADS 1 effectively rules out recurrence and requires routine follow-up. NI-RADS 2 has moderate recurrence rates and requires further evaluation. NI-RADS 3 has a high predictive value for recurrence and necessitates immediate attention.

## Introduction

Head and neck cancers

Head and neck cancers remain a major global health concern originating from the oral cavity, pharynx, larynx, thyroid, and salivary glands [1,2]. The staging of head and neck cancers is performed according to the TNM classification. Squamous cell carcinoma is the most common histopathological type of cancer in this group of neoplasms, with a special relation to the oral cavity, pharynx, and larynx [3]. Tobacco, alcohol, betel quid chewing, and human papillomavirus are considered risk factors in oral and oropharyngeal cancers [4]. The management of head and neck cancers is mainly based on the TNM staging system. Stage I and II cancer treatment is mostly surgical or radiation therapy, while stage III and IV cancers are treated by surgery, radiation therapy, and chemotherapy [5,6]. Other factors that may aid in the treatment are the patient’s condition and the location of the tumor [7,8].

The need for post-treatment surveillance of locoregional recurrence and its impact on survival

The risk of locoregional recurrence in patients treated for head and neck cancer poses a significant threat to patient survival and quality of life. Early detection of locoregional recurrence is vital, as it can significantly influence treatment decisions and improve outcomes [9]. The study conducted by Van Hoe and Hermans [6] highlights that the mean time of detection for recurrent or metastatic disease is approximately 11.5 months, which is well beyond the initial post-treatment imaging assessments typically conducted within the first three to six months. Thus, guidelines advocating for systematic imaging surveillance beyond the initial follow-up are essential to improving long-term survival outcomes. Studies indicate that post-treatment surveillance should extend for at least 2 years to effectively monitor patients, as prolonged follow-up can lead to timely interventions that may halt or reverse disease progression. Hence, the integration of routine imaging into follow-up care is not only a clinical necessity but also a supportive measure for enhancing patient reassurance and coping strategies post-treatment [10].

Complexities of detecting recurrent/residual lesions

Detecting recurrent or residual lesions post-treatment poses several challenges, largely attributed to anatomical distortion and soft tissue changes caused by initial therapies. Surgical interventions, radiation, and chemotherapy can lead to significant alterations in the head and neck anatomy, complicating the interpretation of follow-up imaging studies. Residual lesions may be obscured by post-treatment inflammation or fibrosis, leading to diagnostic dilemmas that can delay appropriate management [5]. The study by Melariri et al. [7] indicates that the prevalence of locoregional recurrence in head and neck cancer patients is alarmingly high, with a reported rate of 15.4%. However, detecting these recurrences can be impeded by the overlapping of post-treatment changes with potential disease processes. Advanced imaging techniques, such as PET-CT, have shown promise in enhancing detection rates compared with conventional modalities, yet the inherent complexities in imaging interpretation remain a challenge for clinicians [11]. 

NI-RADS: a solution for surveillance challenges

NI-RADS is a structured reporting framework developed by the American College of Radiology (ACR) in order to enhance the quality and consistency of imaging reports for patients undergoing surveillance after treatment for head and neck cancers. Developed to address the complexities associated with post-treatment imaging, NI-RADS provides a standardized lexicon that simplifies the communication of imaging findings between radiologists and clinicians. This is crucial given the intricate anatomical and pathological changes that can occur following treatment, which often complicate the interpretation of imaging studies [12]. In the context of head and neck cancer, the post-treatment landscape is characterized by a range of changes, including surgical alterations, radiation effects, and potential disease recurrence. Traditional reporting methods can lead to ambiguities, misinterpretations, and inconsistent management decisions. NI-RADS mitigates these challenges by categorizing findings into a systematic reporting structure that emphasizes clarity. Each category (NI- RADS 1 through NI-RADS 4) offers specific definitions and criteria that assist radiologists in assessing the likelihood of residual disease or recurrence, thus facilitating more informed clinical decision-making and improving patient outcomes [12]. Research has demonstrated that the NI-RADS scoring system shows high inter-reader and intra-reader agreement, indicating that multiple radiologists can consistently apply the criteria to arrive at similar conclusions regarding imaging findings. For instance, a study by Strauss et al. [11] reported almost perfect inter-reader agreement for NI-RADS categorization of primary lesions and lymph nodes. The significance of NI-RADS extends beyond mere categorization; it fosters a collaborative environment where radiologists and oncologists can engage in a more effective dialogue regarding patient care. By providing a common language, NI-RADS helps bridge the gap between imaging interpretation and clinical management, ultimately enhancing the overall quality of care for patients with head and neck cancers [12,13].

NI-RADS categories and their significance

NI-RADS classifies imaging findings into four distinct categories based on the likelihood of malignancy, with each category guiding the subsequent clinical management. NI-RADS 1 indicates benign or expected post-treatment changes and recommends routine follow-up imaging. NI-RADS 2 suggests low suspicion findings, for which direct visual inspection is advised, while NI-RADS 2b denotes intermediate concern and warrants close follow-up imaging within three to six months using CT or PET-CT. NI-RADS 3 represents a high suspicion for residual or recurrent disease and necessitates tissue sampling for definitive diagnosis. NI-RADS 4 confirms disease progression or recurrence, for which initiation of appropriate treatment is recommended [14].

Key NI-RADS-based imaging features for CT

In the assessment of primary tumor site recurrence, NI-RADS 1 corresponds to benign post-treatment changes such as fibrosis, mild soft-tissue thickening, and non-enhancing scar tissue. NI-RADS 2 indicates indeterminate findings and is subdivided into NI-RADS 2a, characterized by non-mass-like focal mucosal enhancement, and NI-RADS 2b, which shows non-mass-like, ill-defined deep soft-tissue changes with only mild differential enhancement. NI-RADS 3 represents a high suspicion of recurrence and is defined by the presence of an enhancing mass with irregular margins at the tumor bed, while NI-RADS 4 indicates definite recurrence, characterized by a large expansile mass with necrosis and aggressive local invasion.

For nodal recurrence in neck assessment, NI-RADS 1 denotes the absence of abnormal lymph nodes and includes expected post-treatment changes. Treated pathological nodes showing central low density, rim enhancement, or necrosis are also categorized as NI-RADS 1. NI-RADS 2 is assigned to newly identified mildly enlarged lymph nodes without necrosis or extranodal extension. NI-RADS 3 indicates a higher likelihood of nodal recurrence, characterized by newly enlarged nodes with central necrosis and possible extranodal spread. NI-RADS 4 represents definite nodal recurrence, marked by large, progressively enlarging necrotic lymph nodes with aggressive features [15].

## Materials and methods

Details of the study

The study was conducted for two years from June 2023 to June 2025. It included a total of 64 patients who were referred to the Department of Radiology at Vydehi Institute of Medical Sciences and Research Centre, Bangalore, for contrast-enhanced computed tomography (CECT) of the head and neck as part of post-treatment follow-up for cancer. The recommended follow-up usually comprises an initial scan within 3 months after successful completion of treatment, followed by every 6-12 months for 2 years, after which the patient is considered to be in remission, assuming all the tests are negative. The study population comprised biopsy-proven head and neck cancer patients who had completed treatment and were older than 18 years. Patients with renal impairment, known allergy to contrast media, pregnant women, and those with newly diagnosed head and neck cancers were excluded from the study. Statistical analysis was performed using the chi-square test to calculate p-values, and a p-value of less than 0.05 was considered statistically significant for all analyzed parameters.

CT Protocol

Multidetector CT was performed on all the patients satisfying the inclusion criteria using Siemens Somatom Definition AS 128 slice Multi-detector CT scanner with 5 mm collimation, gantry speed of 0.05 sec, pitch of 0.8 sec, 120 kvP and 200 effective mAs. The analysis was performed on the axial cuts (5 mm) and thin sections (1 mm) of coronal, axial, and sagittal reconstruction. Based on imaging findings and comparison with prior imaging, NI-RADS categories were allotted for the primary site and nodal site by one head and neck radiologist, and a management plan was recommended. The results were followed up and correlated with the consequent histopathological examination report, follow-up imaging, and clinical examination.

Illustrative cases

Case 1

A 36-year-old male patient, a known case of carcinoma glottis, status post radiotherapy, presented for follow-up in view of foreign body sensation.

**Figure 1:**
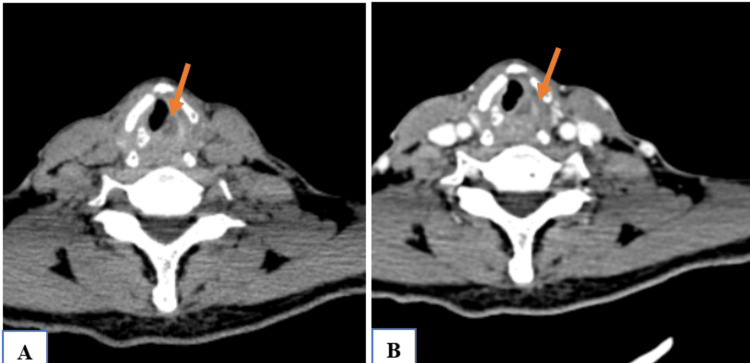
NI-RADS 1 illustrative case (A) Pre-contrast CT: Hypodense thickening of the left glottic region (B) Post-contrast CT: Non-enhancing thickening of the left glottic region, representing normal post-radiation changes (NI-RADS 1) NI-RADS, Neck Imaging Reporting and Data System.

Clinical examination: Indirect laryngoscopy revealed an edematous vocal cord. The patient was suggested routine follow-up every six months (Figure 1).

Case 2 

**Figure 2:**
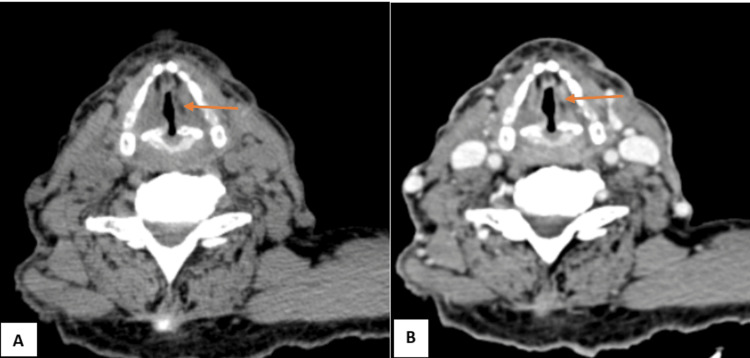
NI-RADS 1 illustrative case (A) Plain CT (axial section) at the level of the vocal cords (B) Contrast-enhanced CT (axial section) at the same level showing diffuse non-enhancing thickening of both true and false vocal cords, causing significant narrowing of the airway, consistent with post-radiation changes (NI-RADS 1). NI-RADS, Neck Imaging Reporting and Data System.

A 79-year-old male patient, a known case of carcinoma supraglottis, status post definitive radiotherapy, presented with difficulty in swallowing and was subjected to CECT head and neck (Figure 2).

Case 3 

**Figure 3:**
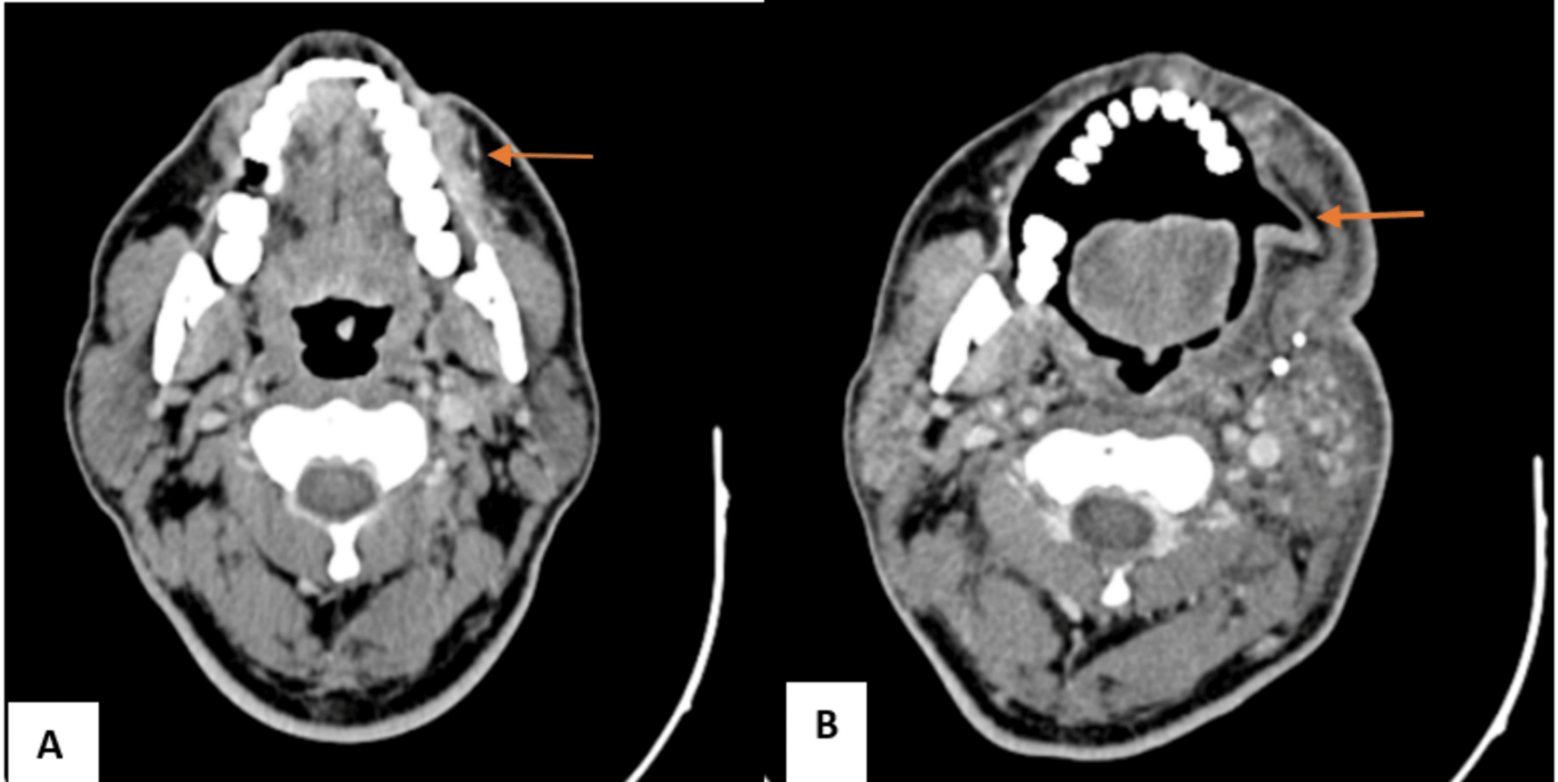
NI-RADS 2a illustrative case (A) Pre-treatment CT: Enhancing lesion in the left buccal space (B) Post-operative CT: Reconstructed flap and adjacent homogenous, mildly enhancing mucosal and subcutaneous thickening noted in the left buccal mucosa, with non-visualization of the left hemimandible and left maxilla (NI-RADS 2a) NI-RADS, Neck Imaging Reporting and Data System.

A 51-year-old male patient, a known case of carcinoma of the left buccal mucosa, status post wide local excision, underwent left segmental mandibulectomy and anterolateral thigh muscle flap, with concurrent chemoradiotherapy, and presented for follow-up CECT head and neck (Figure 3).

Case 4

**Figure 4:**
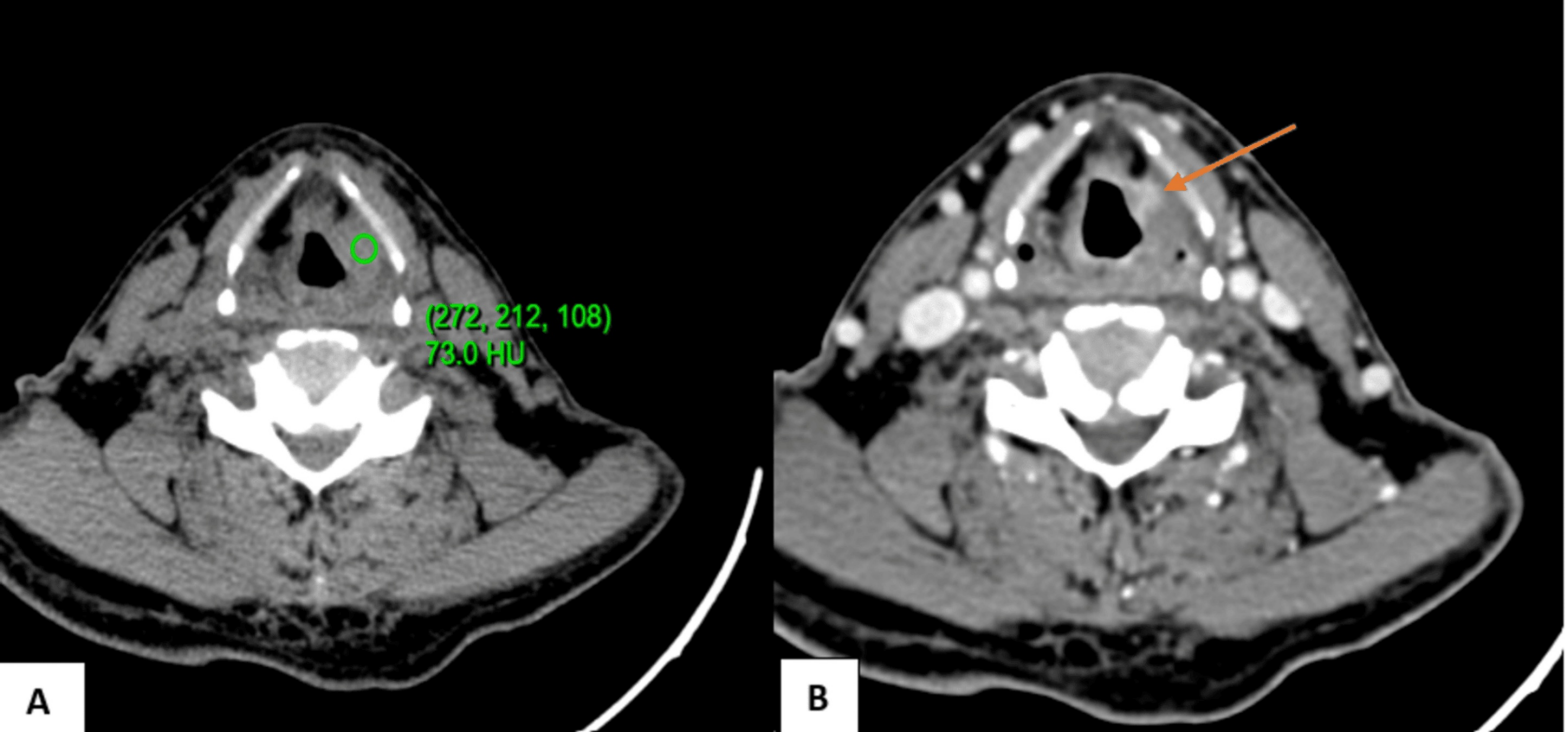
NI-RADS 2b illustrative case (A) Plain axial CT at the level of the true vocal cords (B) Contrast axial CT at the level of the true vocal cords Ill-defined non-mass-like soft tissue thickening with mild post-contrast enhancement (pre-contrast: 73 and post-contrast: 91) in the left vocal cord (NI-RADS 2b) The patient was advised for a follow-up scan after three months. NI-RADS, Neck Imaging Reporting and Data System.

**Figure 5:**
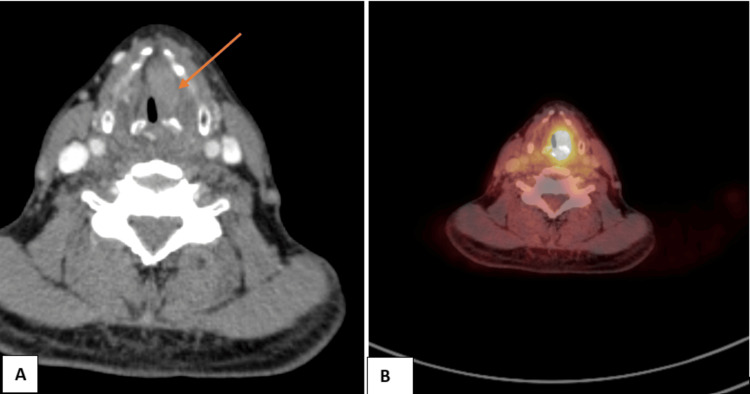
NI-RADS 2b follow-up PET-CT scan (A) Contrast-enhanced CT showing an increase in size of the previously ill-defined enhancing lesion in the left vocal cord, causing narrowing of the airway. (B) FDG PET scan of the same patient as mentioned in case 4 showed increased FDG uptake on a follow-up scan. Hence, it was upgraded to NI-RADS 3 on follow-up. FDG, fluorodeoxyglucose; NI-RADS, Neck Imaging Reporting and Data System.

Follow-up imaging was performed in a 51-year-old male with laryngeal squamous cell carcinoma after chemoradiotherapy. Figure 4 shows the initial follow-up scan, and Figure 5 shows the three-month follow-up scan.

Case 5

**Figure 6:**
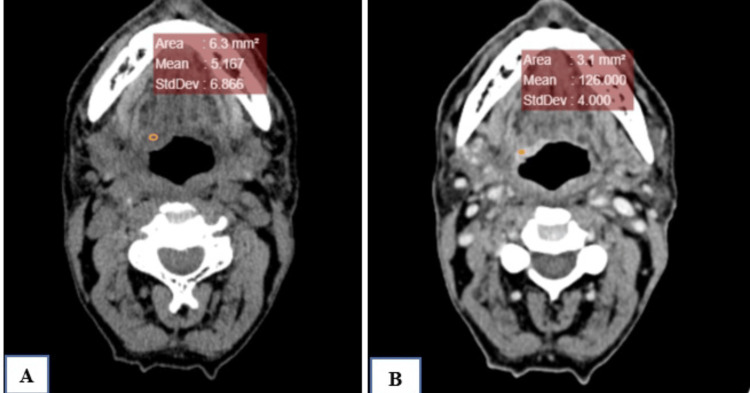
NI-RADS 3 illustrative case A: Axial plain image B: Contrast axial image Findings: A fairly well-defined heterogeneously enhancing lesion in the right vallecula and right pyriform fossa, NI-RADS 3 primary site NI-RADS, Neck Imaging Reporting and Data System.

A 66-year-old male, a known case of carcinoma right pyriform fossa, status post CTRT, presented with a complaint of throat pain (Figure 6). 

Case 6 

**Figure 7:**
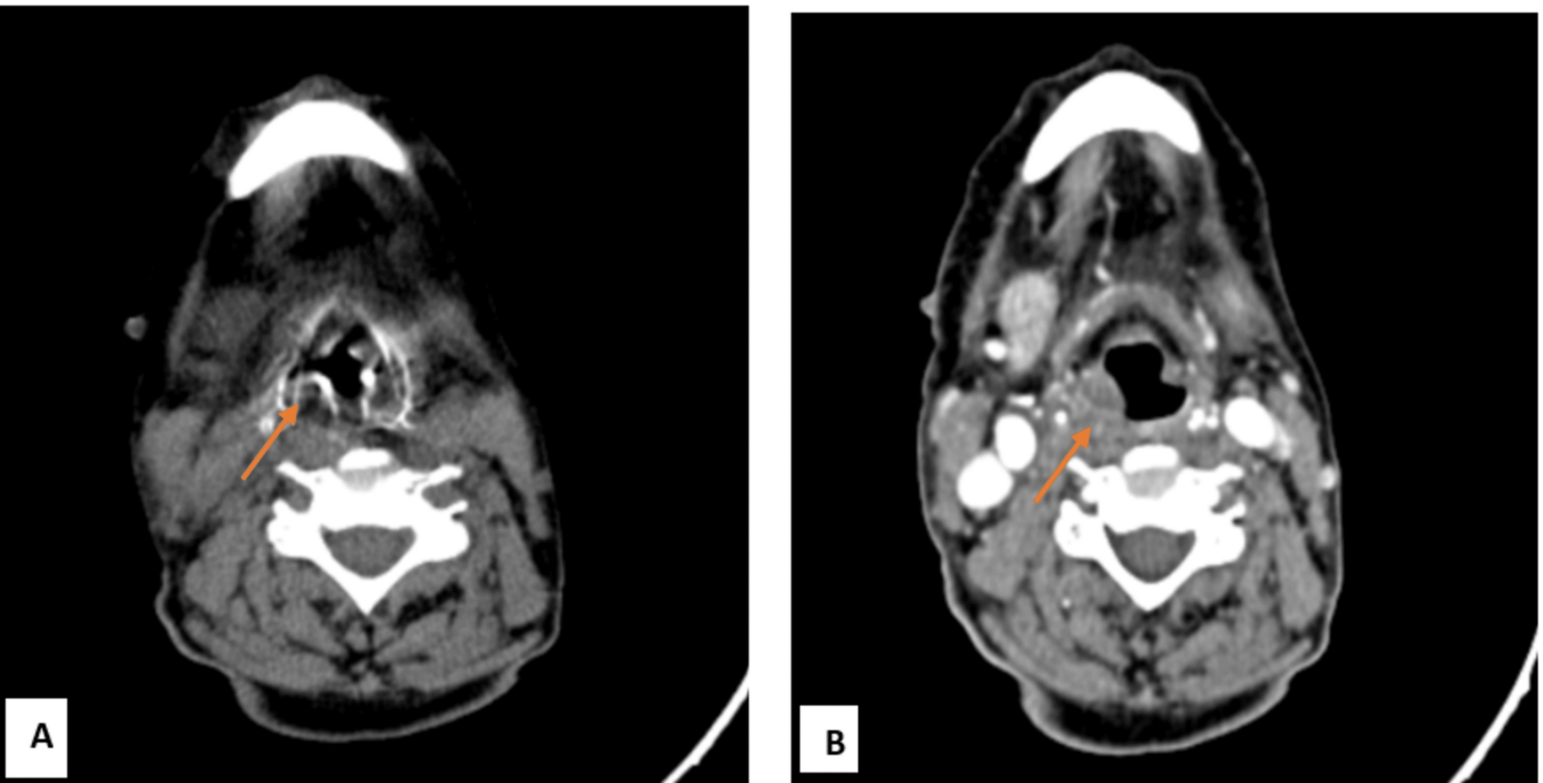
NI-RADS 3 illustrative case (A) Plain axial CT: Well-defined hypodense lesion (pre-contrast HU: 56) in the right tonsillar fossa (B) Contrast axial CT: Well-defined lesion with moderate post-contrast enhancement (post-contrast HU: 80) in the right tonsillar fossa, NI-RADS 3 primary site HU, Hounsfield unit; NI-RADS, Neck Imaging Reporting and Data System.

A 50-year-old female, previously a known case of carcinoma right tonsil, status post CTRT, presented for follow-up and was subjected to CECT head and neck (Figure 7).

Case 7 

**Figure 8:**
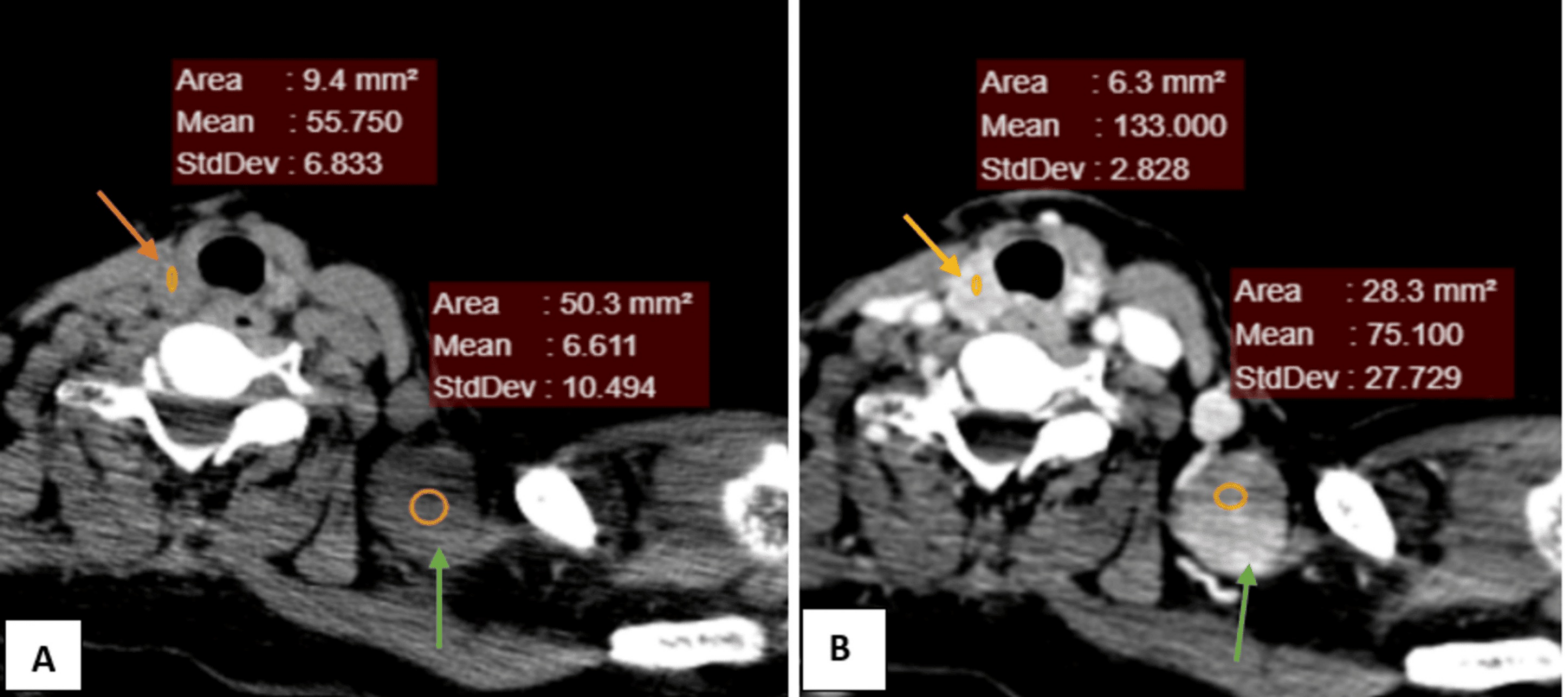
NI-RADS 2 node + NI-RADS 3 primary site (A) Plain CT axial section at the level of the thyroid gland (B) Contrast-enhanced CT axial section Findings: Two well-defined lesions with significant post-contrast enhancement (pre-contrast HU: 55 and post-contrast HU: 133) in the right and left paratracheal region (orange arrow), NI-RADS 3 primary site Another well-defined round lesion with significant post-contrast enhancement (pre-contrast HU: 6 and post-contrast HU: 75) in the left supraclavicular region (green arrow). No areas of necrosis noted, NI-RADS 2 for node HPE report: Positive for nodal recurrence and negative for primary site recurrence HPE, histopathological examination; HU, Hounsfield unit; NI-RADS, Neck Imaging Reporting and Data System.

A 48-year-old female, a known case of multinodular goiter with follicular neoplasm, status post total thyroidectomy, presented with swelling in the left side of the neck and was subjected to CECT head and neck (Figure 8).

Case 8

**Figure 9:**
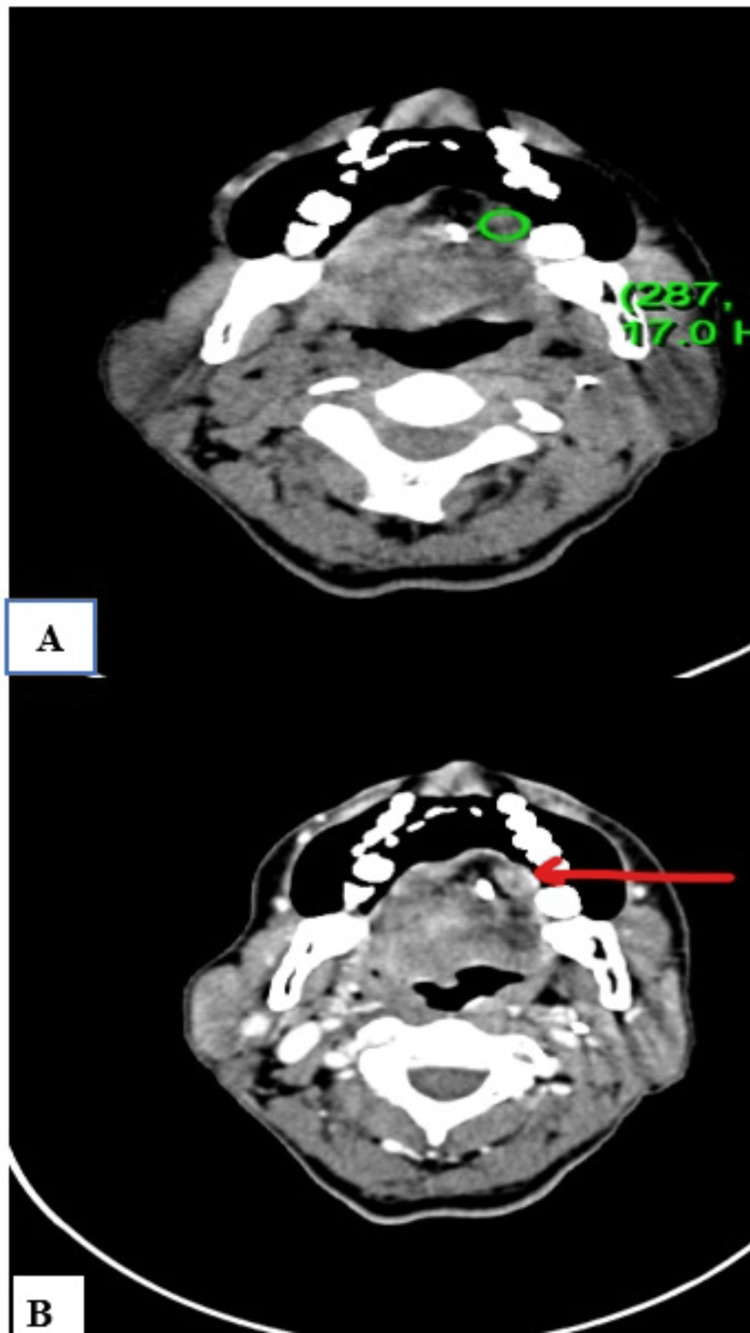
False-positive NI-RADS 3 case (A) Plain axial CT (B) Contrast axial CT Well-defined nodular enhancing lesion (pre-contrast HU: 17 and post-contrast HU: 101) along the lateral border of the flap. The case was labeled as NI-RADS 3 primary site. However, the HPE report revealed no evidence of malignant cells. HPE, histopathological examination; HU, Hounsfield unit; NI-RADS, Neck Imaging Reporting and Data System.

A 54-year-old male patient, a known case of carcinoma of the floor of mouth, status post composite resection with free flap insertion, presented with a complaint of a new growth over the flap site (Figure 9).

## Results

The study comprised a total of 64 patients who were known cases of any head and neck malignancies and came for post-treatment imaging with CECT during the study period (Table 1).

**Table 1:** Result table, including 64 cases CA, carcinoma; HPE, histopathological examination; NI-RADS, Neck Imaging Reporting and Data System.

S. no.	Age	Gender	Type of CA	Primary site	Node	Result
1	76	M	Oral cavity (tongue)	NI-RADS 2	NI-RADS 1	Negative for primary site on follow-up
2	54	M	Oral cavity (tongue)	NI-RADS 3	NI-RADS 1	HPE: negative for primary site
3	49	M	Oral cavity (buccal CA)	NI-RADS 1	NI-RADS 1	Routine follow-up
4	50	F	Oral cavity (buccal CA)	NI-RADS 1	NI-RADS 1	Routine follow-up
5	59	F	Oral cavity (buccal CA)	NI-RADS 1	NI-RADS 1	Routine follow-up
6	42	M	Oral cavity (buccal CA)	NI-RADS 3	NI-RADS 2	HPE: negative for primary site, node remained stable on follow-up
7	48	F	Oral cavity (buccal CA)	NI-RADS 2	NI-RADS 3	HPE: positive for node, negative for primary site
8	40	M	Glottic CA	NI-RADS 2	NI RADS 1	Clinical examination: negative
9	51	F	Oral cavity (buccal CA)	NI-RADS 3	NI-RADS 2	HPE: positive for primary site
						HPE: negative for node
10	18	M	Nasopharyngeal CA	NI-RADS 3	NI-RADS 3	HPE: positive for primary site
						HPE: positive for node
11	70	M	Oropharynx (tonsil CA)	NI-RADS 1	NI-RADS 1	Routine follow-up
12	28	M	Oral cavity (tongue)	NI-RADS 2	NI-RADS 1	Clinical examination: negative
13	59	M	Oral cavity (tongue)	NI-RADS 3	NI-RADS 1	HPE: positive for primary site
14	68	M	Oral cavity (tongue)	NI-RADS 1	NI-RADS 1	Routine follow-up
15	43	M	Oral cavity (buccal CA)	NI-RADS 1	NI-RADS 1	Routine follow-up
16	79	M	Oropharynx	NI-RADS 1	NI-RADS 1	Routine follow-up
17	64	M	Glottic CA	NI-RADS 1	NI-RADS 1	Routine follow-up
18	64	M	Oropharynx	NI-RADS 1	NI-RADS 1	Routine follow-up
19	71	F	Oral cavity (buccal CA)	NI-RADS 3	NI-RADS 1	HPE: positive for primary site
20	48	F	Thyroid CA	NI-RADS 3	NI-RADS 2	HPE: positive for node, negative for primary site
21	61	M	Glottic CA	NI-RADS 1	NI-RADS 1	Routine follow-up
22	51	M	Oropharynx	NI-RADS 1	NI-RADS 1	Routine follow-up
23	49	M	Nasopharynx CA	NI-RADS 1	NI-RADS 1	Routine follow-up
24	50	F	Oropharynx	NI-RADS 3	NI-RADS 2	HPE: positive for primary site, negative for node
25	66	F	Oral cavity (buccal CA)	NI-RADS 1	NI-RADS 1	Routine follow-up
26	55	M	Oral cavity (tongue)	NI-RADS 3	NI-RADS 1	HPE: positive for primary site
27	50	M	Hypopharynx CA	NI-RADS 1	NI-RADS 1	Routine follow-up
28	54	F	Hypopharynx CA	NI-RADS 1	NI-RADS 1	Routine follow-up
29	53	F	Oral cavity (buccal CA)	NI-RADS 1	NI-RADS 2	HPE: negative for node
30	43	F	Oral cavity (buccal CA)	NI-RADS 1	NI-RADS 1	Routine follow-up
31	51	M	Oral cavity (buccal CA)	NI-RADS 2	NI-RADS 1	Clinical examination: negative
32	50	F	Hypopharynx CA	NI-RADS 1	NI-RADS 1	Routine follow-up
33	55	M	Oral cavity (buccal CA)	NI-RADS 1	NI-RADS 1	Routine follow-up
34	36	M	Oral cavity (tongue)	NI-RADS 1	NI-RADS 1	Routine follow-up
35	79	M	Hypopharynx	NI-RADS 1	NI-RADS 1	Routine follow-up
36	61	M	Oral cavity (buccal CA)	NI-RADS 3	NI-RADS 1	HPE: negative for primary site
37	63	M	Oral cavity (buccal CA)	NI-RADS 1	NI-RADS 1	Routine follow-up
38	48	F	Oral cavity (tongue)	NI-RADS 3	NI-RADS 3	HPE: positive for primary site
39	34	M	Oral cavity (tongue)	NI-RADS 1	NI-RADS 1	Routine follow-up
40	39	M	Oral cavity (buccal CA)	NI-RADS 1	NI-RADS 1	Routine follow-up
41	41	M	Hypopharynx	NI-RADS 2b	NI-RADS 1	Clinical inspection: negative, PET-CT: positive
42	57	F	Oral cavity (buccal CA)	NI-RADS 1	NI-RADS 1	Routine follow-up
43	64	M	Oral cavity (buccal CA)	NI-RADS 3	NI-RADS 1	HPE: negative for primary site
44	63	M	Oral cavity (tongue)	NI-RADS 3	NI-RADS 1	HPE: positive for primary site
45	62	F	Oral cavity (buccal CA)	NI-RADS 1	NI-RADS 3	HPE: positive for node
46	56	F	Oral cavity (buccal CA)	NI-RADS 1	NI-RADS 2	Follow-up: negative for node
47	50	M	Glottic CA	NI-RADS 3	NI-RADS 1	HPE: positive for primary site
48	40	F	Oral cavity (buccal CA)	NI-RADS 3	NI-RADS 1	HPE: positive for primary site
49	39	M	Oral cavity (buccal CA)	NI-RADS 3	NI-RADS 1	HPE: negative for primary site
50	55	M	Glottic CA	NI-RADS 1	NI-RADS 1	Routine follow-up
51	51	M	Glottic CA	NI-RADS 1	NI-RADS 1	Routine follow-up
52	53	M	Glottic CA	NI-RADS 1	NI-RADS 1	Routine follow-up
53	50	M	Oral cavity (buccal CA)	NI-RADS 1	NI-RADS 1	Routine follow-up
54	66	M	Hypopharynx CA	NI-RADS 3	NI-RADS 3	HPE: positive for primary site and node
55	59	F	Oral cavity (buccal CA)	NI-RADS 1	NI-RADS 1	Routine follow-up
56	30	M	Oral cavity (buccal CA)	NI-RADS 3	NI-RADS 1	HPE: positive for primary site
57	68	M	Oral cavity (buccal CA)	NI-RADS 1	NI-RADS 1	Routine follow-up
58	37	M	Glottic CA	NI-RADS 1	NI-RADS 1	Clinical examination: negative
59	58	M	Oral cavity (buccal CA)	NI-RADS 3	NI-RADS 3	HPE: positive for primary site and node
60	63	M	Glottic CA	NI-RADS 2	NI-RADS 1	Clinical examination: negative
61	52	M	Oral cavity (buccal CA)	NI-RADS 1	NI-RADS 1	Routine follow-up
62	26	F	Thyroid CA	NI-RADS 3	NI-RADS 1	HPE: negative for primary site
63	73	M	Parotid CA	NI-RADS 3	NI-RADS 1	HPE: negative for primary site
64	46	M	Parotid CA	NI-RADS 1	NI-RADS 1	Routine follow-up

Distribution of the study group based on location

Buccal carcinoma was the most common head and neck cancer, observed in 28 patients (43.75%), followed by tongue and glottic cancers. Nasopharynx, thyroid, and parotid were the least common types of head and neck cancers (Table 2).

**Table 2:** Distribution of study group based on location

Type of cancer	Count	Percentage (%)
Oral cavity (buccal)	28	43.75
Oral cavity (tongue)	10	15.62
Glottic	9	14.06
Hypopharynx	6	9.38
Oropharynx	5	7.81
Nasopharynx	2	3.12
Thyroid	2	3.12
Parotid	2	3.12

Distribution of the NI-RADS category for the primary site and lymph nodes

The majority of the cases were allotted NI-RADS category 1 for evaluation at the primary site, which constituted 36 cases (56.25%), followed by NI-RADS 3 (32.81%), and the least were NI-RADS 2 (10.94%). The majority of cases were allotted NI-RADS category 1 for evaluation of the lymph node status, which constituted 52 cases (81.25%). NI-RADS 2 and 3 had an equal distribution of six cases each (Table 3).

**Table 3:** Distribution of NI-RADS categories for the primary site and lymph node status in the study NI-RADS, Neck Imaging Reporting and Data System.

NI-RADS category	Primary site	Percentage (%)	Nodal status	Percentage (%)	
NI-RADS 1	36	56.25	52	81.25	
NI-RADS 3	21	32.81	6	9.38	
NI-RADS 2	7	10.94	6	9.38	

Performance outcomes for NI-RADS at the primary site

Overall, tumor recurrence occurred in 14 out of 64 patients at the primary site, with a recurrence rate of 21%. The recurrence rate for each NI-RADS category was 0% for NI-RADS 1, 14.29% for NI-RADS 2, and 61.90% for NI-RADS 3. The p-value is 3.05 × 10⁻⁷ (<0.001), which is highly statistically significant. This confirms a strong association between the NI-RADS category and recurrence risk (Table 4).

**Table 4:** Performance outcomes for NI-RADS categories at the primary site NI-RADS, Neck Imaging Reporting and Data System.

NI-RADS category	Total cases	Outcome positive	Positive (%)	Outcome negative	Negative (%)
NI-RADS 1	36	0	0.00	36	100.00
NI-RADS 2	7	1	14.29	6	85.71
NI-RADS 3	21	13	61.90	8	38.10
Total	64	14	21.88	50	78.12

Predictive value of NI-RADS 2 at the primary site

Table 5 shows that NI-RADS 2 has low sensitivity (7.14%) but high specificity (88%), meaning it is better at ruling out recurrences than detecting them.

**Table 5:** Predictive value of NI-RADS 2 at the primary site NI-RADS, Neck Imaging Reporting and Data System.

Metric	Value (%)
Positive predictive value	14.29
Negative predictive value	77.19
Sensitivity	7.14
Specificity	88

Performance outcomes for the NI-RADS 3 category of the primary site

This shows that NI-RADS 3 has the following performance statistics: (1) High sensitivity (92.86%): It indicates that it is very effective at detecting true recurrences, meaning very few actual recurrences go undetected. (2) Moderate positive predictive value (61.9%): It indicates that around 38% of cases flagged as recurrence turn out to be false positives. (3) High negative predictive value (97.67%): If NI-RADS 3 suggests no recurrence, it is very reliable, with only a 2.33% chance of missing an actual recurrence. (4) Good specificity (84%): It effectively rules out non-recurrence cases but still has some false positives (Table 6).

**Table 6:** Performance outcomes for NI-RADS 3 at the primary site

Metric	Value (%)
Positive predictive value	61.9
Negative predictive value	97.67
Sensitivity	92.86
Specificity	84

Overall performance outcomes for the NI-RADS categories for nodal recurrence

The overall recurrence of lymph nodes was 8 out of the total 64 patients, indicating a 12% recurrence. The recurrence rate for each NI-RADS category was 0% for NI-RADS 1, 33.33% for NI-RADS 2, and 100% for NI-RADS 3. The p-value for the association between NI-RADS categories (1, 2, and 3) for nodal recurrence outcomes is 5.62 × 10⁻¹², which is extremely small. A p-value this low (p < 0.001) indicates a highly significant association between NI-RADS categories for nodal recurrence. This means that the probability of recurrence is strongly dependent on the NI-RADS classification. NI-RADS 3 has the highest recurrence rate, followed by NI-RADS 2, while NI-RADS 1 has no recurrence cases (Table 7).

**Table 7:** Overall performance outcomes for NI-RADS categories for nodal recurrence NI-RADS, Neck Imaging Reporting and Data System.

NI-RADS category (nodes)	Total cases	Outcome positive (%)	Outcome negative (%)	
NI-RADS 1	52	0 (0)	52 (100)	
NI-RADS 2	6	2 (33.33)	4 (66.67)	
NI-RADS 3	6	6 (100)	0 (0)	
Total	64	8 (12.50)	56 (87.50)	

## Discussion

In this study, 64 patients with a history of head and neck cancer undergoing surveillance imaging were categorized into NI-RADS 1, 2, and 3 based on their post-treatment imaging. The majority of cases were classified as NI-RADS 1 (36 cases, 56.25%), followed by NI-RADS 3 (21 cases, 32.81%), and the least were NI-RADS 2 (7 cases, 10.94%). The overall recurrence rate at the primary site was 21.88% (14/64 cases).

A strong correlation was observed between NI-RADS categorization and recurrence rates. NI-RADS 1 demonstrated a 0% recurrence rate, confirming its high negative predictive value (100%). This suggests that patients classified as NI-RADS 1 require routine follow-up with no immediate need for biopsy. NI-RADS 2 exhibited a recurrence rate of 14.3%, indicating a moderate risk, while NI-RADS 3 had a significantly higher recurrence rate of 61.9%. The chi-square test yielded a p-value of 3.05 × 10⁻⁷, indicating a highly statistically significant association between NI-RADS classification and recurrence at the primary site.

Performance of NI-RADS in predicting nodal recurrence

When evaluating lymph node recurrence, NI-RADS 1 nodes (52 cases) showed 0% recurrence, reinforcing their reliability in predicting negative outcomes. NI-RADS 2 nodes had a recurrence rate of 33.3% (2/6 cases), while all NI-RADS 3 nodes (6/6 cases) were recurrent (100%). The p-value for nodal recurrence (5.62 × 10⁻¹²) further confirms the statistical significance of NI-RADS classification in nodal recurrence prediction.

The high sensitivity (100%) and specificity (96.55%) of NI-RADS 3 nodes suggest that this category is highly reliable for detecting true recurrence cases. In contrast, NI-RADS 2 showed a lower positive predictive value (33.33%), indicating that one-third of cases classified as NI-RADS 2 were indeed recurrent, but two-thirds were false positives. These findings emphasize the need for careful clinical correlation and additional confirmatory tests (e.g., biopsy) for NI-RADS 2 cases.

Recurrence based on primary tumor site

The recurrence rates across different primary tumor sites varied significantly. The highest number of NI-RADS 3 cases was observed in oral cavity cancers, with variable recurrence rates. Despite these differences, the p-value for recurrence across primary sites was 0.577, indicating that while NI-RADS is strongly predictive overall, the specific location of cancer does not independently influence recurrence rates in this dataset.

Overall performance and clinical implications

The study confirms that NI-RADS 3 is highly effective in detecting recurrences, with a positive predictive value of 61.9% for the primary and 100% for nodal recurrence. The negative predictive value of NI-RADS 1 remains at 100%, reassuring clinicians that a NI-RADS 1 classification reliably indicates a low risk of recurrence.

The findings also suggest that NI-RADS 2 remains a gray zone, where careful evaluation and further diagnostic steps are necessary. The lower PPV (33.33%) of NI-RADS 2 for nodal recurrence and the moderate recurrence rate at the primary site (14.3%) indicate that some cases categorized as NI-RADS 2 may progress to recurrence, necessitating individualized follow-up strategies.

The findings of the current study align well with previous research, confirming NI-RADS as a reliable tool for predicting recurrence in head and neck cancers. The study reported a 61.9% recurrence rate for NI-RADS 3, which is slightly lower than Abdelrahman et al. (80.8%) [16] and Baba et al. (74.4%) [17], but closely matches Lee et al. (65%) [18]. NI-RADS 2 had a lower recurrence rate (14.3%) compared with other studies (25%-29%), suggesting possible differences in sample size, post-treatment changes, or imaging interpretation. The 0% recurrence rate for NI-RADS 1 is consistent with global findings, reaffirming its strong negative predictive value. The high sensitivity (92.86%) of NI-RADS 3 in this study confirms its ability to detect recurrences effectively, although its positive predictive value was slightly lower (61.9%), indicating some overestimation of risk. The statistical significance (p < 0.001) across all studies further validates NI-RADS as a valuable, standardized framework for post-treatment surveillance, helping clinicians optimize follow-up strategies and reduce unnecessary interventions. However, larger multicenter studies could further refine its accuracy, especially in distinguishing NI-RADS 2 and 3 cases.

Limitations

Due to the limited sample size of only seven cases of NI-RADS 2 in the evaluation of the primary site, there is a limitation to assess the statistical power.

## Conclusions

This study reinforces the utility of NI-RADS as a reliable tool for risk stratification in head and neck cancer surveillance. The high statistical significance (p<0.001) in both primary site and nodal recurrence underscores its predictive accuracy. NI-RADS 1 effectively rules out recurrence and requires routine follow-up. NI-RADS 2 has moderate recurrence rates and requires further evaluation. NI-RADS 3 has a high predictive value for recurrence and necessitates immediate attention.

Future studies with larger sample sizes and multimodal imaging comparisons could further enhance the robustness of NI-RADS as a universal risk assessment tool in oncology practice. By integrating NI-RADS into routine surveillance, clinicians can optimize patient management, reduce unnecessary biopsies, and improve early detection of recurrent disease.
